# Basic psychological need satisfaction of collegiate athletes: the unique and interactive effects of team identification and LMX quality

**DOI:** 10.3389/fspor.2024.1342995

**Published:** 2024-05-02

**Authors:** Joé G. Leduc, Frédéric Boucher, Dominic L. Marques, Eric Brunelle

**Affiliations:** ^1^ Department of Management, John Molson School of Business, Concordia University, Montreal, QC, Canada; ^2^ Department of Management, Laval University, Quebec, QC, Canada; ^3^ Pôle Sports, HEC Montréal, Montreal, QC, Canada

**Keywords:** basic psychological needs, student athletes, team identification, coach-athlete relationship, LMX

## Abstract

**Purpose:**

The present study sought to understand the relationships between team identification, leader-member exchange (LMX) quality, and the basic psychological need satisfaction of collegiate athletes, as well as the moderating role of coach-athlete LMX quality.

**Methods:**

Self-reported data from 319 collegiate athletes were analyzed using SPSS version 29. The relationships between the study variables were tested by moderation analysis using PROCESS macro model 1.

**Results:**

Regression analyses showed team identification to be positively related to the satisfaction of the needs for competence and relatedness, while LMX quality was positively related to the satisfaction of the needs for competence and autonomy. Furthermore, moderation analyses showed that LMX quality positively moderated the relationship between team identification and the satisfaction of the needs for competence and relatedness.

**Conclusion:**

The results of this study highlight the important role that team identification and LMX quality play in the satisfaction of the basic psychological needs of collegiate athletes. The implications of these results for the optimal functioning of collegiate athletes are discussed.

## 1 Introduction

Student athletes must cope with a unique combination of demands and expectations. On the one hand, they have to deal with sport-related stressors such as performance pressure, fatigue, and injuries. On the other hand, they must also manage the demands and the workload associated with their academic position (1, 2). This situation forces them to excel in both areas in order to maintain their student athlete status (3). As a consequence of these challenges, mental health and well-being issues are particularly prevalent in the student athlete population [e.g., (4–7)]. More precisely, research has shown that in university environments, the prevalence of athletes living with a mental health problem can reach 18% (3). Moreover, according to Åkesdotter et al. (8), 50% of athletes could experience a mental health issue during their career. These findings highlight the importance of understanding the factors that contribute to the optimal functioning of student athletes.

On that matter, previous studies have consistently shown that the satisfaction of student athletes’ basic psychological needs (BPNs) for competence, autonomy, and relatedness plays a key role in terms of promoting their well-being, their performance, their motivation, and their personal growth (9–11). BPNs are described by Ryan et al. as “essential psychological nutrients for individuals’ adaptation, integrity, and growth” [(12) p. 1]. Moreover, the satisfaction of these three BPNs has also been negatively related to stress and injuries (12), and to athlete burnout (13–15). While past studies have contributed to clarifying the benefits of BPN satisfaction for student athletes, our understanding of the social factors that relate to the satisfaction of those needs is still very limited (16). To address this important gap, the present study draws from self-determination theory [SDT; (11, 17)], and more specifically from basic psychological needs theory [BPNT; (18)] to investigate the social factors that contribute to the BPN satisfaction of student athletes. Importantly, BPNT posits that social environments can facilitate or hinder the satisfaction of BPNs. Considering that social relationships play a pivotal role in the sport context (19, 20) and that the team and the coach are crucial relational targets for student athletes, our study aims at better understanding the relationships between these two social factors and the satisfaction of the BPNs of student athletes. Specifically, we examine the relationships between athletes’ level of team identification (21) and of coach-athlete relationship quality, as captured by leader-member exchange [LMX, (22, 23)], and the level of satisfaction of their BPNs.

While the relation between team identification and BPN satisfaction has not been examined among student athletes, research has shown that teammates are crucial social agents in facilitating athletes’ fulfillment of these needs (24, 25). Team identification as defined by Ashforth and Mael (21), refers to the extent to which an individual derives their sense of self from belonging to a team, reflecting their level of connection to that team. In this regard, Greenaway et al. (26) found that social identity gain promoted the satisfaction of the global psychological needs for control, self-esteem, belonging and meaning, while social identity loss thwarted the satisfaction of these needs. These results suggest that student athletes who have greater levels of identification with their team may experience greater need satisfaction. Despite the significance of social identification in sport psychology (27), the specific association between student athletes’ level of team identification and the satisfaction of their BPNs for competence, autonomy and relatedness remains unexplored.

Regarding coaches, it appears that the coach-athlete relationship is also crucial in the sport context (28) in terms of creating a positive social environment for athletes (29). Congruently, Chu & Zhang's (30) review of 20 studies shows that positive social environments fostered by coaches and peers are positively related to athletes’ satisfaction of their BPNs. In this regard, leader-member exchange (LMX) theory examines the dyadic relational quality between a leader and a member (31). Chen et al. (32) theorized that LMX quality promotes outcomes which significantly overlap with the satisfaction of BPNs, namely, employees’ perception of competence and choice. Consistently, manager-employee LMX quality was found to be positively related to the satisfaction of the BPNs for competence, autonomy and relatedness of employees (33). BPN satisfaction was also found to mediate the relationship between LMX quality and well-being among counselors (34). Due to the relational similitudes between the manager-employee and the coach-athlete relationships, similar relations are expected to emerge in the sport context (35). Nonetheless, the relationship between coach-athlete LMX quality and the satisfaction of the BPNs of student athletes is yet to be investigated.

Moreover, coach-athlete LMX quality has yet to be investigated as a moderator of the relationship between team identification and the satisfaction of the BPNs of student athletes. Indeed, since the coach-athlete relationship is embedded within the team context, it may be that the strength of the relationship between team identification and the BNP satisfaction of student athletes depends on the level of LMX quality of the coach-athlete relationship. In response to these shortcomings, the present study examines the unique and interactive relationships of team identification and LMX quality with the satisfaction of the three BPNs of student athletes.

This study makes important contributions to the sport psychology literature. First, by examining the relationship between team identification and the BPNs of student athletes, this study deepens our understanding of the influence of the team as a key social factor promoting the satisfaction of the BPNs of student athletes. Moreover, by exploring the relationship between LMX quality and the satisfaction of the BPNs of student athletes, this study furthers our knowledge of the role of coach-athlete relationship quality in the optimal functioning of student athletes. Lastly, by examining LMX quality as a moderator of the relationship between team identification and BPN satisfaction, this study uncovers the interactive effect of these two key social factors in facilitating the satisfaction of the BPNs for competence, autonomy, and relatedness of student athletes. Practically, our research may help coaches and sports organizations identify the actions they can take to promote the optimal functioning of their student athletes.

### 1.1 Theoretical background and hypotheses

Self-determination theory is a key theory to understand human motivation and functioning (11). As a metatheory, SDT encompasses six mini-theories, one of which is basic psychological needs theory. According to BPNT, needs are universal and essential nutriments for optimal functioning, and individuals must satisfy three BPNs in order to experience growth, integrity and well-being. First, the need for competence corresponds to the need to feel effective and capable in one's interactions with the environment so as to achieve desired outcomes. Second, the need for autonomy is the need to feel free to choose and organize one's activities and behaviors, as well as endorsing one's decisions and behaviors as coming from oneself. Third, the need for relatedness refers to a need to feel close to others, be part of a group and have reciprocal relationships characterized by respect, care, and support (10, 36–38). In this regard, a key source of satisfaction of these needs is the social environment individuals find themselves in (11, 18).

### 1.2 Team identification and basic psychological need satisfaction

According to social identity theory (39), one’s social identity corresponds to “that part of an individual’s self-concept which derives from his/her knowledge of his/her membership of a social group (or groups) together with the value and emotional significance attached to that membership”. [(40) p. 255] By extension, social identification is the perception of being one with a group of people (e.g., a team, an organization). Such identification leads to behaviors which are congruent with one's social identity, as well as support for activities and organizations which exemplify it. In light of this, we argue that student athletes’ level of team identification will positively influence the satisfaction of their BPNs.

First, regarding the satisfaction of the need for competence, when student athletes perceive that they are key members of their team and that this membership is personally significant to them, they are likely to act and feel in accordance with the norms and values of the team. Integrating parts of a group's values and goals into their self-concept also intensifies the impact of group experiences on individual outcomes (41), such as personally experiencing the successes and failures of their team (42). Thereby, greater team identification is likely to lead student athletes to experience the accomplishments of their team more strongly and personally. In this regard, SDT predicts that such perceived accomplishments will provide student athletes with greater opportunities to feel effective and competent (18). Furthermore, social identification with a group promotes a sense of embeddedness within one's social network and an increase in social support from the group, resulting in greater self-efficacy beliefs (43). Although perceived competence and self-efficacy beliefs are theoretically and statistically distinct constructs, self-efficacy is strongly and positively related to perceived competence in the context of physical exercise (44). Hence, student athletes who identify strongly with their team are likely to feel more embedded within and supported by their social network, which is likely to promote the level of encouragement and positive feedback they receive. According to SDT, such feedback promotes the satisfaction of the need for competence (18).

Second, when it comes to the need for autonomy, previous research shows that identification with a group leads to a higher likelihood of thinking and acting in terms of membership with the group, as well as support for activities associated with it (21, 45). In light of this, student athletes who strongly incorporate their team membership into their sense of self may internalize team concordant goals and behaviors. In this regard, SDT posits that the perception of choice and internal initiation of behavior is key to the satisfaction of the need for autonomy (18). Thus, student athletes who are highly identified with their team may perceive their decisions and behaviors within their sports team as more self-endorsed, resulting in greater satisfaction of their need for autonomy. Conversely, student athletes who do not identify strongly with their team may experience the demands and expectations of their team as sources of control, resulting in lower satisfaction of their need for autonomy.

Third, in terms of the need for relatedness, past scholarship shows that group identification increases group cohesion, cooperation, pro-social behavior and positive outlook on the group (46, 47). Hence, student athletes who strongly identify with their team are more likely to feel valued by their teammates, thereby facilitating the emergence of reciprocal relationships and close social ties. According to SDT, this social connectedness is likely to promote the satisfaction of the BPN for relatedness (18). Moreover, social identification is posited to lead to a sense of belongingness and unity with the group (48) Thereby, student athletes who strongly identify with their team are likely to experience increased satisfaction with their social relations as well as greater feelings of belongingness with their team, resulting in heightened satisfaction of their need for relatedness. In line with this reasoning, we posit:

*H1: Team identification is positively related to the satisfaction of the need for (a) competence, (b) autonomy, and (c) relatedness.*

### 1.3 Leader-member exchange quality and basic psychological need satisfaction

Another key social factor in the sport context is the coach. Indeed, the coach-athlete relationship can have an important influence on student athlete outcomes (25, 49–51). In this regard, leader-member exchange (LMX) theory is a model of leadership which examines the quality of the dyadic relationship between a leader and a follower (22, 23). LMX posits that leadership is a partnership between two individuals which develops over time. This relation is initially characterized by contractual, formal and hierarchical exchanges. Then, it matures towards exchanges which transcend self-interest and which are based on reciprocal influence (8). In a high-quality exchange, the leader provides key resources and the member gives support, resulting in a mutually benefiting relationship. In a low-quality exchange, the member does not have access to such important resources and is given fewer opportunities from their leader (52). On this matter, in a social context characterized by a high coach-athlete LMX quality, SDT predicts that the BPN satisfaction of athletes will be supported (18).

Indeed, coach autonomy support has been found to predict BPN satisfaction in multiple sports contexts (53–55). Moreover, coach-athlete relationship characteristics such as quality, interdependence, and rapport have also been positively related to the satisfaction of the BPNs of athletes (25, 49–51). These related concepts are similar to LMX quality in that they capture the reciprocal and non-contractual characteristics of coach-athlete relationships. Thus, based on SDT and past findings, we argue that coach-athlete LMX quality is positively related to the satisfaction of the BPNs of student athletes.

First, SDT states that to satisfy their need for competence, student athletes must perceive that they are effective in influencing their environment (18). This influence often takes the form of contributing to the success of their team, progressing towards valued goals, and overcoming difficulties. On that matter, SDT states that positive feedback is crucial to the perception of being effective in impacting one's environment (18). As the main figure of authority and leadership in the team, the coach is the most important source of such opportunity and feedback for student athletes. In this respect, high LMX quality relationships are likely to be characterized by superior performance feedback due to their high levels of trust. Moreover, previous work has shown that opportunities to perform are crucial to the perceived sense of competence of student athletes (56). On this point, high quality exchanges provide opportunities for development since leaders in these relations encourage and support followers to engage in challenging tasks (57). Furthermore, since high LMX quality relations are characterized by liking and professional respect (58), they are likely to provide optimal levels of challenges (33). Lastly, leaders engaged in high LMX quality relationships also share positive expectations of superior performance (59) and provide mastery experiences (60), which are also likely to bolster the student athlete's sense of competence. Thus, we argue that greater LMX quality is likely to promote the satisfaction of the need for competence of student athletes.

Second, when it comes to the need for autonomy, SDT posits that individuals must perceive themselves as being able to freely make decisions and to act them out. They need to perceive that they have a choice and that their behavior is self-initiated in order to satisfy this need (18). Since the coach is the formal authority figure in the sport context, he/she is an important source of autonomy or control for the student athlete. In high quality LMX relationships, leaders tend to reduce control and provide more opportunities for members to engage in the decision-making process (33, 61, 62), which is likely to increase the satisfaction of their need for autonomy. Moreover, due to the trust which is characteristic of high LMX quality relations (58) the coach is likely to give more independence to their athlete. Conversely, the impersonal and contractual nature of low LMX quality relations is likely to be experienced by student athletes as stifling their ability to make meaningful decisions and as making them more dependent on their coach's will, thus reducing their perceived autonomy.

Third, SDT states that to satisfy the need for relatedness, individuals need to perceive that they have close and satisfying social ties (18). Consistently, high LMX quality relations are characterized by reciprocal interactions, as well as social and emotional support to followers (33, 63). Characteristic properties of high-quality LMX relationships such as obligation and trust (58) are also closely related to the caring and respectful relations which tend to satisfy the need for relatedness (36). Accordingly, high quality LMX relationships are likely to bolster student athletes’ experience of closeness and reciprocity with their coach, promoting the satisfaction of their need for relatedness. Such relations may also provide student athletes with greater access to the coach's social network (64), leading to more stable and satisfying relations with other members of the organization. Conversely, low LMX quality relations produce impersonal and transactional relations which are not likely to generate close and caring relations, resulting in lower satisfaction of student athletes’ need for relatedness. Based on SDT and previous findings, we posit:

*H2: LMX quality is positively related to the satisfaction of the need for (a) competence, (b) autonomy, and (c) relatedness.*

### 1.4 The moderating effect of LMX quality

As previously discussed, the team and the coach of student athletes are two key social factors influencing the satisfaction of their BPNs. Moreover, due to the embeddedness of the coach within the team, it may be the case that these factors interact in predicting BPN satisfaction. Indeed, SDT states that multiple elements of the social context can support or thwart need satisfaction within a given situation (18). For example, a study by Fraina (65) found positive interactions between the coach and teammates in predicting the BPN satisfaction of athletes. In line with this finding, we argue that when student athletes have a high-quality LMX relationship with their coach, the positive association between team identification and the satisfaction of their BPNs will be amplified. Conversely, when student athletes have a low-quality LMX relationship with their coach, the strength of the relationship between team identification and need satisfaction will be dampened. In other words, we posit that coach-athlete LMX quality moderates the relationship between team identification and the satisfaction of the BPNs of student athletes.

Considering the need for competence, when team identification is high, student athletes may experience their team's successes as coming from themselves (42). However, if LMX quality is low, the coach may not provide the challenges and opportunities to fully take part and internalize the team's victories (33). Moreover, in low quality relationships, the coach may not provide sufficient levels of feedback and personal recognition, which are key for student athletes’ perception of being part of team success. Following SDT logic, this situation is likely to reduce student athletes’ perception that they are effective in their sport and that they are actively contributing to their team. In other words, low quality LMX relations are likely to dampen the influence of team identification on the satisfaction of the need for competence. Conversely, when LMX quality is high, opportunities to take part in team victories, performance feedback, and recognition from the coach are likely to amplify the influence of high team identification on the satisfaction of the need for competence by making team successes even more salient and personally experienced.

Regarding the need for autonomy, high team identification may increase student athletes’ perception of volition in their team since they perceive their behaviors as more consistent with their sense of self. Nonetheless, if LMX quality is low, the coach is likely to exercise more authority and control due to low levels of trust, thus dampening the perception of volition stemming from student athletes’ identification with their team. According to SDT, this presence of control is likely to reduce the perceived autonomy of the athlete (18). On the other hand, if LMX quality is high, greater levels of trust are likely to reduce the level of control exercised by the coach, promoting student athletes’ perception that their behaviors in the team context are self-endorsed, thus amplifying the impact of high team identification on the satisfaction of their need for autonomy.

**Figure 1:**
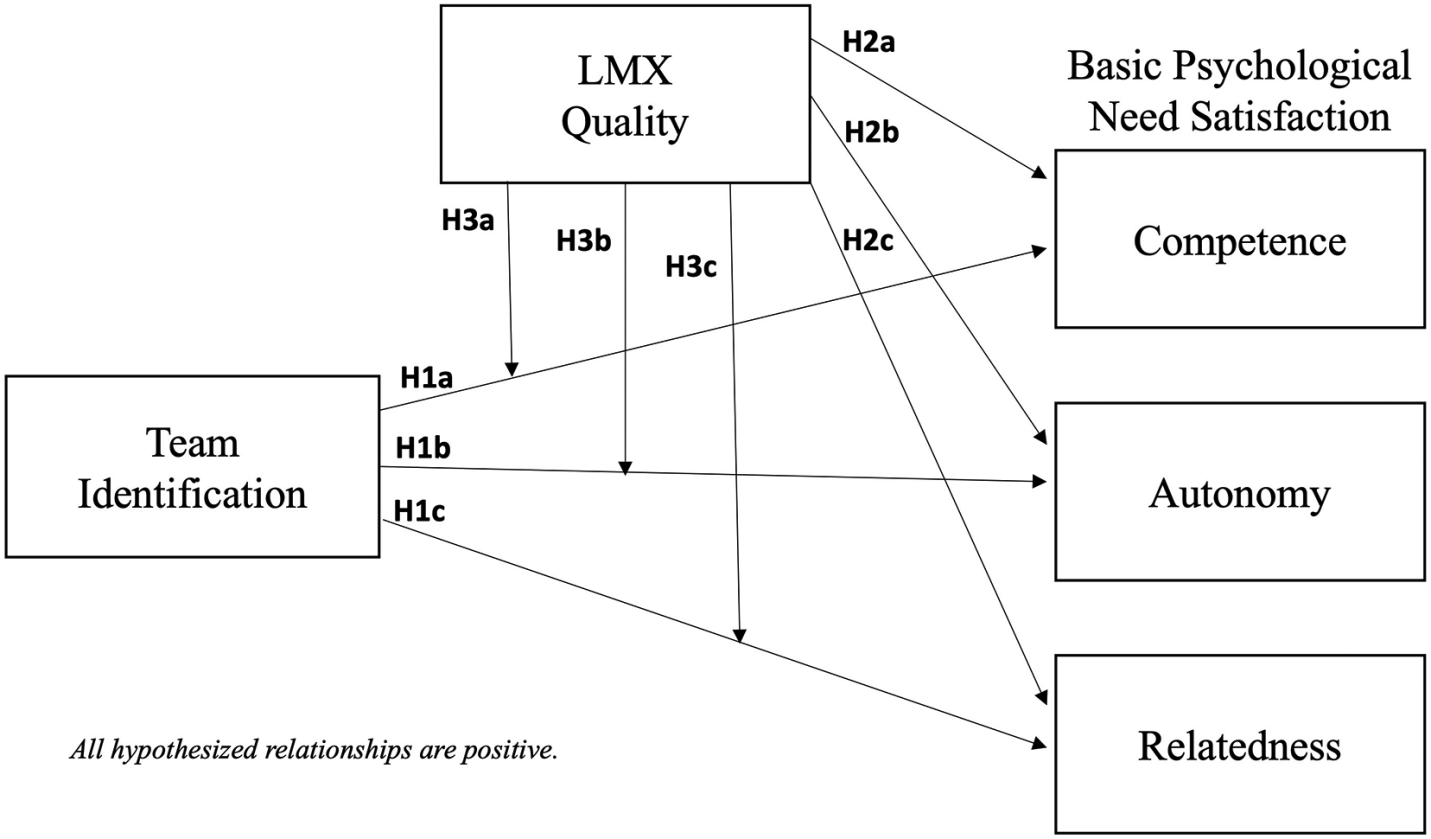
Hypothesized research model.

Considering the need for relatedness, high team identification may foster student athletes’ sense of belongingness (48) and of being valued by their teammates, both of which are predicted by SDT to promote perceived relatedness (18). However, if LMX quality is low, student athletes may not benefit from the social-emotional support of the coach (63, 64), reducing their perception of being an important member of their team, and dampening the influence of team identification on the satisfaction of their need for relatedness. Conversely, if LMX quality is high, support from the coach is likely to promote student athletes’ perception of being socially valued and accepted in their team, thereby amplifying the influence of high team identification on the satisfaction of their need for relatedness. Thus, as shown in Figure 1, we posit:

*H3: LMX quality positively moderates the relationship between team identification and the satisfaction of the need for (a) competence, (b) autonomy, and (c) relatedness, such that this relationship is strengthened when LMX quality is high and weakened when it is low.*

## 2 Method

### 2.1 Participants and procedures

Respondents were recruited to participate in this study via email by their sports department administrator. In terms of eligibility, participants had to be enrolled as collegiate-level athletes at the time of data collection. Only participants who met this criterion received an email invitation to participate in the study. The email contained a short description of the study and a link to the online questionnaire. The informed consent form and our survey were published on the platform Qualtrics. Participants were informed that their participation was completely voluntary and that their answers would remain anonymous. All information that would compromise respondent anonymity was removed from the dataset prior to data analysis. Data collection started in June 2022 and concluded in March 2023. In this period, around 1,000 collegiate athletes received an invitation to participate. Overall, our response rate was 32%.

Our sample consisted of 319 student athletes from two major universities. Student athletes were part of teams in the following sports: track and field (14.4%), swimming (11.6%), rugby (9.7%), soccer (9.7%), volleyball (9.7%), cheerleading (6.3%), badminton (5.6%), tennis (5.6%), basketball (5.6%), football (5.3%), golf (3.4%), cross-country (3.1%), alpine skiing (2.5%), triathlon (2.2%), other (2.2%), hockey (1.6%) and cross-country skiing (1.3%). Athletes were part of masculine (*n = 92,* 29%), feminine (*n = 137, 43%*), and mixed sports teams (*n = 90, 28%*). The average team tenure was 2.4 years (*SD *= 1.4). The coach-athlete relationship spanned 2.4 years on average (*SD *= 1.5). Cross-sectional data was obtained through an online questionnaire including 28 items. Participants were asked to rate each item on a Likert scale ranging from 1 = strongly disagree to 7 = strongly agree.

### 2.2 Measures

Table 1 reports the internal consistency of measures. Based on Cronbach’s alpha, all measures show adequate reliability.

**Table 1:** Means, standard deviations, correlations, and reliability indices.

Variable	*M*	*SD*	1	2	3	4	5	6	7	8
1. Team tenure	2.37	1.44	–							
2. Coach-athlete dyadic tenure	2.41	1.45	.60*	–						
3. Team identification	6.12	.89	.05	.16*	(.78)					
4. LMX quality	5.63	1.33	-.08	.09	.38*	(.96)				
5. Basic psych. need satisfaction	5.44	.76	.05	.18*	.56*	.61*	(.84)			
6. Competence	5.12	1.02	.14	.17*	.38*	.41*	.75*	(.77)		
7. Autonomy	5.18	1.15	-.01	.13*	.29*	.62*	.78*	.32*	(.82)	
8. Relatedness	6.02	.86	-.04	.11*	.63*	.29*	.71*	.35*	.33*	(.85)

*M* and *SD* are used to represent mean and standard deviation, respectively. Cronbach's alphas are presented in parentheses on the diagonal.

* *p* < .05.

#### 2.2.1 Team identification

We adapted the four-item social identification (FISI) scale to assess team identification (66). More precisely, two items are adapted from Doosje et al. (67): “I feel committed to my sports team” and “I identify with my sports team”. The two others are “I am glad to be part of my sports team”, and “Being part of my sports team is an important part of how I see myself” (68).

#### 2.2.2 Leader-member exchange quality

We adapted the seven-item Leader-Member Exchange 7 Questionnaire (69) to measure the quality of the dyadic relationship between the coach and the student athletes. To do so, we changed the referent in the original measure, which was the “immediate supervisor”, to the “coach”. The items were also adapted to fit with the sports context and to facilitate the understanding of items by respondents (i.e., student-athletes). The adapted items are (1) “In general, I know where I stand with my coach”, (2) “My coach understands my problems and my needs”, (3) “My coach recognizes my achievements and my potential”, (4) “My coach is personally inclined to help me solve problems in my sport practice”, (5) “I can count on my coach to support me when I need it”, (6) “My coach has enough confidence in me that he/she would defend and justify my decisions if I were not present to do so” and (7) “The interactions with my coach are effective”. The phrasing of items was adapted to fit with the Likert agreement scale used throughout our questionnaire. Specifically, interrogative items such as “How well do you feel that your immediate supervisor recognizes your potential?” were transformed into declarative items “My coach recognizes my achievements and my potential”. Confirmatory factor analysis was conducted in Mplus (version 8.7) using the MLR estimator. The one-factor LMX quality measure showed excellent fit based on CFI, TLI and SRMR, and marginally acceptable fit based on RMSEA [*χ*^2^(14) = 57.196, *p *< .001, CFI = .968, TLI = .953, RMSEA = .098, SRMR = .024].

The questions relating to the two measures above were translated from English to French by two members of our research team who are fluent in both languages. The first author translated the items from English to French. The fourth author compared the items to ensure that their meaning did not change. The translated items were then tested for accuracy in a small subsample of the target population before their administration in the main study.

#### 2.2.3 Basic psychological need satisfaction

We used the 15-item satisfaction of fundamental needs in sports scale [*l’échelle de satisfaction des besoins fondamentaux en contexte sportif*; (16)] to assess the satisfaction of the BPNs for competence, autonomy, and relatedness. Each subscale included five items. Items include “In my sport, I feel free to make choices”, “In my sport, I do not feel very competent” (reversed), and “In my sport, I feel at ease with others”.

#### 2.2.4 Control variables

Our first control variable is the number of years each collegiate athlete has been part of their sports team (i.e., team tenure). We also controlled for the number of years each collegiate athlete has been in relation with their coach (i.e., coach-athlete dyadic tenure). In past research, tenure is controlled based on researchers’ objectives. For example, Chaudhry et al. (70) controlled manager organizational tenure and employee-manager dyadic tenure to determine the level of alignment of perceived LMX between employees and managers. In our case, team tenure can impact student athletes’ level of identification with their team, as well as the satisfaction of their BPNs. For coach-athlete dyadic tenure, the length of the relationship can impact LMX quality (71).

### 2.3 Statistical analysis

In this study, all statistical analyses were performed using SPSS 29.0. Preliminary analyses and tests of hypotheses were conducted as follows. First, descriptive statistics were performed to highlight the characteristics of the sample. Second, principal component analysis using explained variance and correlation analysis using Pearson's coefficient were conducted to detect common method bias. Third, Cronbach's alpha values were computed to assess the reliability of our measures. Descriptive statistics were also conducted to examine the means and standard deviations of study variables and correlation analysis was performed to identify the relations between them. Fourth, the variance inflation factor was calculated to test for the presence of multicollinearity between variables. Fifth, tests of hypotheses were performed through moderation analyses using Process Macro model 1 (72). Team identification was used as the independent variable, LMX quality was used as the moderator, and the satisfaction of the BPNs for competence, autonomy and relatedness were used as the dependent variables. The statistical significance level of all tests was set at *p *< .05.

## 3 Results

### 3.1 Common method variance

Since we are using cross-sectional, single-source data, we assessed common method bias before testing our hypotheses. This was done to verify if the variance of our study variables was true variance, or if it was due to common measurement method. First, we conducted Harman's single factor test (73). This technique uses exploratory factor analysis in which variables are constrained so that there is no rotation. According to Podsakoff et al. (74), if the single factor or one general factor explains more than 50% of the variance, common method bias is present. Results of principal component analysis in SPSS 29.0 revealed that 5 distinct factors from 26 items accounted for 69% of the total variance. The first unrotated factor captured 37% of the variance in our data. Hence, no single factor accounted for most of the variance in the data.

Second, we used the correlation matrix procedure to determine common method bias. According to Bagozzi et al. (75), a substantial correlation (*r* ≥ .90) among principal constructs indicates common method bias. By examining the principal constructs in our correlation matrix, we identified the strongest association as that of team identification and the satisfaction of the need for relatedness (*r *= .63). Thus, evidence supports the idea that common method bias is not a major issue in our data. Table 1 presents the means, standard deviations, correlation coefficients and reliability indices for our constructs.

### 3.2 Test of hypotheses

Moderation analyses were performed with SPSS 29.0, using Process Macro model 1 (72) and a 95% confidence interval. Team tenure and coach-athlete dyadic tenure were controlled for in our model. Before performing our analyses, we calculated the variance inflation factor (VIF) to test for the presence of multicollinearity. Since the VIF score was lower than 2.5 (VIF = 1.2), we considered that there were no multicollinearity problems (76). The results of these analyses are presented in Table 2.

**Table 2:** Moderation analyses.

	Competence		Autonomy		Relatedness	
	Est.	SE	Est.	SE	Est.	SE
Control variables						
Team tenure	0.16*	0.06	−0.01	0.06	−0.10	0.05
Coach-athlete tenure	0.01	0.06	0.07	0.06	0.07	0.05
Predictors						
Team identification (TI)	0.30*	0.05	0.06	0.05	0.66*	0.05
LMX quality	0.36*	0.05	0.59*	0.05	0.08	0.05
Interaction						
TI × LMX quality	0.11*	0.03	0.00	0.03	0.11*	0.03
*R* ^2^	0.28		0.39		0.43	

Standardized estimates are reported.

* *p* < .05.

As expected, we found team identification to be moderately related to the satisfaction of the need for competence (*β *= .30, (313), 95% CI [0.19, 0.40]), and strongly related to the satisfaction of the need for relatedness (*β *= .66, (313), 95% CI [0.56, 0.75]), supporting H1a and H1c. However, no significant relationship was found between team identification and the satisfaction of the need for autonomy, providing no support for H1b. Also, we found LMX quality to be moderately related to the satisfaction of the need for competence (*β *= .36, (313), 95% CI [0.26, 0.47]) and strongly related to the satisfaction of the need for autonomy (*β *= .59, (313), 95% CI [0.49, 0.68]), supporting H2a and H2b. However, LMX quality was not significantly related to the satisfaction of the need for relatedness, providing no support for H2c. Contrary to H3b, our results did not show LMX quality to significantly moderate the relation between team identification and the satisfaction of the need for autonomy. However, as expected, we found that LMX quality had a small moderation effect on the relationship between team identification and the satisfaction of the need for competence (*β *= .11 (313), 95% CI [0.04, 0.17]). LMX quality also had a small moderation effect on the relationship between team identification and the satisfaction of the need for relatedness (*β *= .11, (313), 95% CI [0.05, 0.17]).

**Figure 2:**
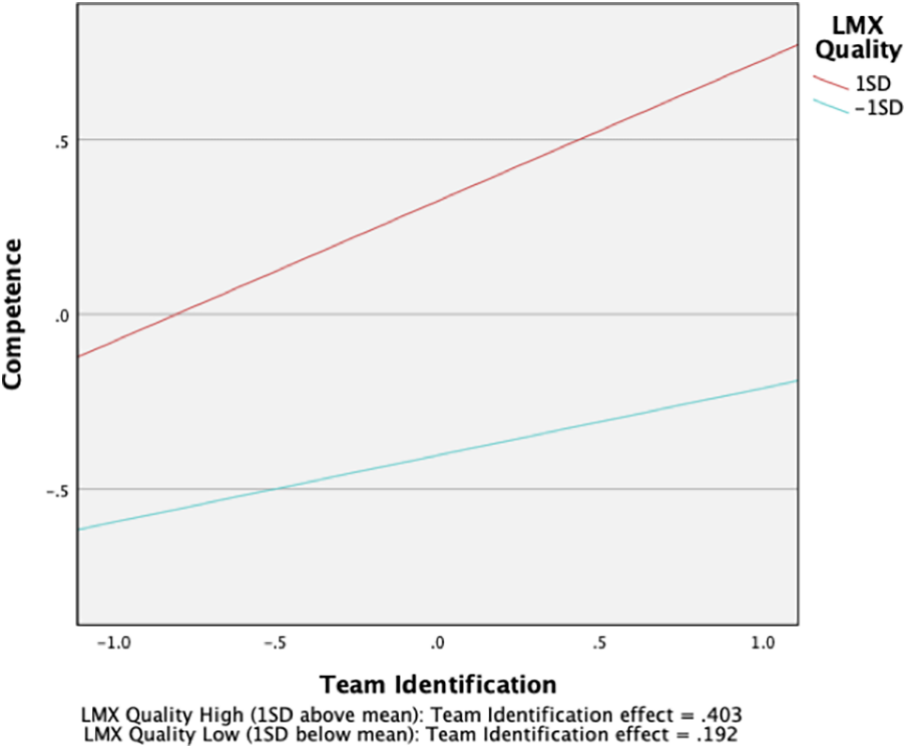
Moderating effect of LMX quality on the team identification-competence need satisfaction relationship.

**Figure 3:**
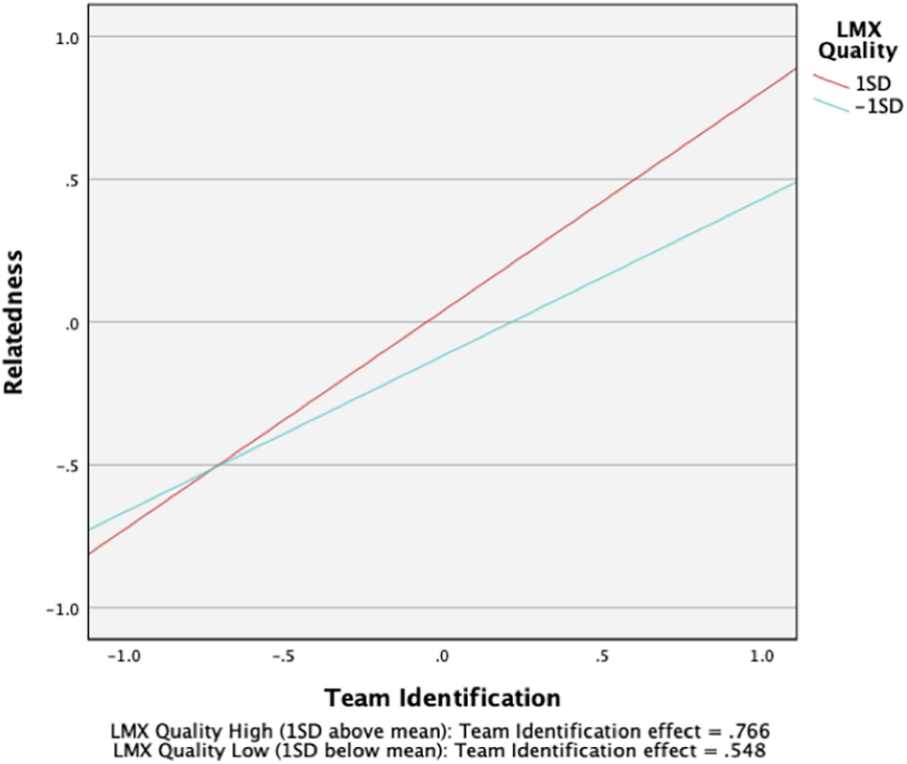
Moderating effect of LMX quality on the team identification-relatedness needs satisfaction relationship.

To fully support H3a and H3c, the form of these interactions should conform to the hypothesized patterns. Therefore, based on recommendations by Cohen et al. (77), the moderating effects were interpreted by plotting the regression equations in relation to two levels of LMX quality, namely, one standard deviation below the mean and one standard deviation above the mean. In line with our expectations, the slope of the relationship between team identification and the satisfaction of the need for competence was stronger for student athletes who had high quality relationships with their coach (*β *= .40 *(313), 9*5% CI [0.26, 0.54]) than for student athletes who had low quality relationships with their coach (*β *= .19 *(310),* 95% CI [0.08, 0.30]). Similarly, the slope of the relationship between team identification and the need for relatedness was stronger for student athletes with high quality LMX relationships (*β *= .77 *(313),* 95% CI [0.64, 0.89]) than for student athletes with low quality LMX relationships *β *= .55 *(313),* 95% CI [0.45, 0.65]). Overall, H3a and H3c are supported by the results of simple slopes analysis and the results depicted in Figures 2, 3, which means that LMX quality exercised a moderating effect on the relationships between team identification and the satisfaction of the needs for competence and relatedness.

## 4 Discussion

The objective of this study was to gain a better understanding of the social factors which contribute to the satisfaction of the BPNs of student athletes. Specifically, we examined the unique and interactive effects of collegiate athletes’ team identification and coach-athlete LMX quality levels on the satisfaction of their BPNs for competence, autonomy and relatedness. Results showed that team identification is positively related to the satisfaction of the needs for competence and relatedness. Furthermore, our results indicated that LMX quality is positively related to the satisfaction of the needs for competence and autonomy. Moreover, results of this study showed that LMX quality positively moderates the relationships between team identification and the satisfaction of the needs for competence and relatedness, so that the strength of these relationships is amplified when LMX quality is high. Using key social factors within student athletes’ environment, our findings suggest that team identification and coach-athlete relationship quality promotes the satisfaction of BPNs. Given that satisfaction of these needs leads to greater well-being, motivation and performance (10, 11), our study contributes to the sport psychology literature by highlighting the importance of student athletes’ social environment for their optimal functioning in the sport context.

### 4.1 Theoretical contributions

Our study makes several key contributions to the discipline of sport psychology. First, despite Rees et al.'s (27) theorizing which highlights social identification as a crucial construct in this field, the relations between team identification and the satisfaction of the three BPNs of student athletes have not been examined in prior research. By showing that team identification significantly relates to the satisfaction of the needs for competence and relatedness among collegiate athletes, our study highlights novel associations with the satisfaction of two BPNs and deepens our understanding of the social factors that promote BPNs satisfaction in the sport context (18, 37). Regarding the nonsignificant relation between team identification and the satisfaction of the need for autonomy, although collegiate athletes strongly identified with their team, this identification was not related to an increased perception that their decisions and behaviors were more volitional. While unexpected, this result aligns with previous findings which showed that the coach, rather than teammates, is the key social factor influencing the autonomy of student athletes (30). Indeed, as the main source of authority in the team, the coach has great influence over athletes’ autonomy. In light of our result, it may be that the increased internalization of goals and expectations resulting from greater team identification does not result in a greater sense of autonomy for student athletes. Nonetheless, team identification appears as a new and important social factor which relates to the satisfaction of the needs for competence and relatedness among student athletes.

Moreover, we extend prior scholarship on LMX quality and the satisfaction of BPNs by uncovering their relations in the new context of college sport. Past research in the work context has found a positive relationship between manager-employee LMX quality and the satisfaction of the BPNs for competence, autonomy and relatedness of employees (33, 34). Our findings now suggest that high LMX quality in coach-athlete relationships also promotes the satisfaction of the needs for competence and autonomy among collegiate athletes. This furthers our understanding of the superior-subordinate relationship as a key social factor in the satisfaction of BPNs in the sport context (37, 78, 79). Contrary to findings in the work domain, the relationship between LMX quality and the satisfaction of the need for relatedness was not significant in our study. This result may be because, even if high LMX relations provide greater socio-emotional support to student athletes (63), the hierarchical distance between the coach and the athletes may hinder the creation of the close ties which are likely to satisfy the need for relatedness of student athletes. This idea is partly consistent with previous research showing that teammates contribute more to the satisfaction of the need for relatedness than the coach (30). Nonetheless, further research is needed to determine the source of the disparity of the associations between LMX quality and the satisfaction of the need for relatedness among employees and student athletes.

Another significant contribution of this study resides in the finding that LMX quality moderates the relationships between team identification and the satisfaction of the BPNs for competence and relatedness of student athletes; a relation that was not examined in prior research. Thus, our study goes beyond highlighting two social factors that promote BPNs satisfaction in the sport context (18, 37) and furthers the application of SDT by showing that complementary social factors have a synergistic effect on the satisfaction of two BPNs among student athletes. Regarding the nonsignificant moderating effect of LMX quality on the relationship between team identification and the satisfaction of the need for autonomy, team identification is not directly related to the satisfaction of the need for autonomy, and it seems that this absence of relation is not conditional on the level of LMX quality of student athletes.

Taken together, our study shows that team identification and coach-athlete LMX quality are both associated with the satisfaction of specific BPNs among student athletes. Notably, neither one of these social factors individually relates to the satisfaction of all three needs; it is only when both team identification and LMX quality are increased that satisfaction of student athletes’ needs for competence, autonomy and relatedness is heightened. In other words, in our model, for student athletes to report increased satisfaction of their three BPNs, they have to report an increase in team identification and in LMX quality with their coach. This study thus provides a deeper understanding of the relations between key social factors and the satisfaction of the BPNs which lead to the optimal functioning of student athletes.

### 4.2 Practical implications

Findings of the present study suggest ways in which coaches and sports organizations may be able to facilitate the satisfaction of the BPNs of collegiate athletes and promote their optimal functioning. Regarding team identification, coaches may encourage and facilitate activities and behaviors which tend to increase collegiate athletes’ identification with their team, such as participating in team-building activities and informal meetings, wearing team-branded clothes, and engaging in shared rituals within the sports practice. A key example of identification promoting behaviors is the ritualistic dance that the All Blacks, the national New Zealand rugby team, engage in before every competition. Student athletes’ joint participation in such practices is likely to amplify team identification, making membership in the team a key part of their identities.

Regarding LMX quality, coaches may promote high quality relations with as many of their collegiate athletes as possible by expressing liking, trust, professional respect and loyalty to each of them (58). Indeed, by transcending contractual exchanges and engaging in reciprocal relationships with student athletes, coaches may increase the satisfaction of their BPNs. Moreover, collegiate athletes themselves may use these findings to promote the satisfaction of their needs for competence, autonomy and relatedness. By participating in the activities and behaviors mentioned above, collegiate athletes can increase identification with their own team. Further, by treating their coach according to the characteristics of high-quality LMX relations, collegiate athletes can initiate a personal and reciprocal relationship with their coach.

### 4.3 Limitations and directions for future research

Like all studies, ours has some limitations. First is the cross-sectional nature of our study. Indeed, all of our study variables were collected at a single point in time, which may have led to common method variance (74). Nonetheless, the single factor test (73) and the correlation matrix procedure to determine common method bias we performed provide evidence that this was not a major issue in our study. Moreover, while we considered team identification and LMX quality as antecedents of BPN satisfaction in our model, we recognize that there may be reciprocal relationships between these constructs. Indeed, increased need satisfaction may lead student athletes to strengthen their relationship with their coach, resulting in greater LMX quality. The satisfaction of the need for relatedness may also increase the tendency of student athletes to identify with their team. Nevertheless, the theoretical grounding of our model gives us good reasons to think that it represents the main directionality of the relationships between our constructs. For future research, a longitudinal design would be warranted to better understand the directionality of these relations.

Furthermore, by focusing on coach-athlete LMX quality as perceived by collegiate athletes, we only explored a fragment of this crucial relationship. To get a better grasp of this dyad, future studies may also examine the perspective of the coach. Indeed, examining this relation from the coaches’ standpoint could deepen our knowledge of hierarchical relationships in college sport. Moreover, our sample specifically focused on collegiate athletes. Future research may examine student athletes at different levels of competition and education to see if the relations we found are consistent across contexts. The importance of team identification for student athletes’ needs satisfaction may be stronger in highly competitive college sport than high-school sports concentration programs. Indeed, due to the highly competitive nature of college sport, the in-group/out-group distinction between one's team and one's opponent team is likely to be greater in our sample than in student athletes at less competitive levels, resulting in higher team identification in our case (M = 6.12).

Lastly, we only examined the individual level of analysis and measurement in this study. Indeed, we argue that each collegiate athletes’ individual level of identification with their team and LMX quality with their coach are most relevant to the satisfaction of their individual BPNs. However, because of the nested nature of our theoretical framework, team identification and LMX quality could also be examined at the team level. Thus, future studies may investigate our conceptual model at multiple levels simultaneously through multilevel analyses. Indeed, the associations between the aggregate level of team identification and the satisfaction of the BPNs of individual athletes within that team could be examined. Moreover, the level of dispersion in LMX quality within a team could also be investigated in relation to the BPN satisfaction of student athletes^1^.

## 5 Conclusion

In conclusion, this study extends our knowledge of the relations between key social factors and the satisfaction of BPNs in the sports context. Specifically, we examined the unique and interactive associations of team identification and LMX quality with the satisfaction of the BPNs for competence, autonomy and relatedness among collegiate athletes. Our results support a model in which collegiate athletes’ level of team identification relates to the satisfaction of their BPNs for competence and relatedness, and their level of coach-athlete LMX quality relates to the satisfaction of their BPNs for competence and autonomy. Our data also supports LMX quality as a positive moderator of the relations between team identification and the satisfaction of the BPNs for competence and relatedness. On the whole, our work extends prior scholarship by highlighting the independent and interactive associations between important social factors and the satisfaction of the BPNs which promote optimal functioning among collegiate athletes.

## Data Availability

The raw data supporting the conclusions of this article will be made available by the authors, without undue reservation.

## Footnotes

^1^We would like to thank an anonymous reviewer for his/her insightful comments on this issue.
